# Prevalence and Prognostic Value of *Myocardial Injury* in the Initial Presentation of SARS-CoV-2 Infection among Older Adults

**DOI:** 10.3390/jcm10163738

**Published:** 2021-08-23

**Authors:** Isabel Arnau-Barrés, Ana Pascual-Dapena, Inmaculada López-Montesinos, Silvia Gómez-Zorrilla, Luisa Sorlí, Marta Herrero, Xavier Nogués, Mila Montero, Olga Vázquez, Natalia García-Giralt, Ramón Miralles, Robert Güerri-Fernández

**Affiliations:** 1 Department of Geriatrics, Hospital del Mar, 08003 Barcelona, Spain; iarnau@psmar.cat (I.A.-B.); mherrero@psmar.cat (M.H.); ovazquez@psmar.cat (O.V.); 2 Departament de Medicina, Universitat Autònoma de Barcelona, 08193 Barcelona, Spain; anapascuald@gmail.com (A.P.-D.); ramon.miralles@uab.cat (R.M.); 3 Facultad de Ciencias de la Salud y de la Vida, Universitat Pompeu Fabra, 08003 Barcelona, Spain; 4 Department of Infectious Diseases, Institute Hospital del Mar of Medical Research, Hospital del Mar, IMIM, 08003 Barcelona, Spain; ilopezmontesinos@psmar.cat (I.L.-M.); sgomezzorrilla@psmar.cat (S.G.-Z.); lsorli@psmar.cat (L.S.); mmontero@psmar.cat (M.M.); 5 Department of Internal MedicineInstitute Hospital del Mar of Medical Research, Hospital del Mar, IMIM, 08003 Barcelona, Spain; xnogues@psmar.cat (X.N.); ngarcia@imim.es (N.G.-G.)

**Keywords:** myocardial injury, older adults, prognosis, SARS-CoV-2

## Abstract

Myocardial involvement during SARS-CoV-2 infection has been reported in many prior publications. We aim to study the prevalence and the clinical implications of acute myocardial injury (MIN) during SARS-CoV-2 infection, particularly in older patients. The method includes a longitudinal observational study with all consecutive adult patients admitted to a COVID-19 unit between March–April 2020. Those aged ≥65 were considered as older adult group. MIN was defined as at least 1 high-sensitive troponin (hs-TnT) concentration above the 99th percentile upper reference limit with different sex-cutoff. Results. Among the 634 patients admitted during the period of observation, 365 (58%) had evidence of MIN, and, of them, 224 (61%) were older adults. Among older adults, MIN was associated with longer time to recovery compared to those without MIN (13 days (IQR 6-21) versus 9 days (IQR 5-17); *p* < 0.001, respectively. In-hospital mortality was significantly higher in older adults with MIN at admission versus those without it (71 (31%) versus 11 (12%); *p* < 0.001). In a logistic regression model adjusting by age, sex, severity, and Charlson Comorbidity Index, the OR for in-hospital mortality was 2.1 (95% CI: 1.02–4.42; *p* = 0.043) among those older adults with MIN at admission. Older adults with acute myocardial injury had greater time to clinical recovery, as well as higher odds of in-hospital mortality.

## 1. Introduction

Myocardial involvement has been reported as one of the clinical presentations of individuals with severe acute respiratory syndrome coronavirus 2 (SARS-CoV-2) infection [1,2]. Cardiological symptoms associated with coronavirus disease-19 (COVID-19) caused by SARS-CoV-2 may include a wide range of presentations from a troponin elevation, reflecting an underlying non-ischemic myocardial injury to a myocardial infarction [3,4]. The diagnosis of myocardial injury (MIN) in hospitalized patients has significantly increased with the widespread use of high-sensitivity troponin (hs-TnT) assay. A precise definition of MIN is needed in order to distinguish it from a myocardial infarction. Whereas myocardial infarction is a form of MIN that requires clinical or electrical evidence of myocardial ischemia and necrosis, MIN is defined as at least 1 cardiac troponin concentration above the 99th percentile upper reference limit [5]. In a large series in New York, by Lala et al., MIN was present in up to 36% of cases [6]. In another large study, MIN was present in 10% of the cases but was associated with significantly worse prognosis and affected more to older individuals [7]. However, data are lacking about the real clinical relevance, especially among older adults, in the current COVID-19 outbreak [8].

It is key to understand whether MIN during the acute COVID-19 episode is a bystander epi-phenomenon or has a relevant clinical role in the outcome of the infection.

A consistent risk for myocardial injury is age [3]. Aging has a well-known detrimental impact on cardiac structure and vasculature. In addition, SARS-CoV-2 infection might cause a worsening of clinical conditions through different mechanisms, such as direct myocardial injury and endothelial binding, T-cell death, and increased inflammation [3]. 

However, no consistent data looking into this specific issue has been reported in older adults with SARS-CoV-2 infection. During the first COVID-19 wave, the largest proportion of patients was over 65 years old [9], making this group of patients an interesting target population. Increasing evidence shows the association between SARS-CoV-2 and MIN, along with increased mortality [2]. Despite being a prevalent and troublesome disease in older adults, there are currently no large studies that deal with the manifestations of COVID-19 in this particular setting. 

On the basis of our clinical observation in our cohort, along with the available evidence, we hypothesized that SARS-CoV-2 might have early effect on the heart of older adults, presenting mainly as MIN in hospitalized individuals. Accordingly, we aim to describe the prevalence and the clinical characteristics and prognosis of MIN as onset manifestation in a large cohort of older patients with confirmed SARS-CoV-2 infection.

## 2. Patients and Methods

This is a retrospective observational study conducted at Hospital del Mar in Barcelona, Spain. This hospital provides healthcare to an area up to 500,000 people. During the first wave of the pandemic, the hospital created a COVID-19 unit that was equipped with 450 beds for in-hospital admission and with 80-beds for critical care. There is an electronic medical record and a centralized registry of all individuals admitted to the COVID-19 unit. For this study, we included all patients admitted to the COVID-19 unit for ≥48 h between 9 March and 1 April 2020. 

Admission criteria to the COVID-19 unit was having a confirmed SARS-CoV-2 infection. This was by having a positive real-time polymerase chain reaction (rt-PCR) for SARS-CoV-2 in nasopharyngeal samples, obtained by trained personnel at hospital admission, and clinical symptoms compatible with SARS-CoV-2 infection (respiratory symptoms, such as dyspnea, cough, sore throat, changes in taste/smell; or uni-/bilateral interstitial infiltrates in chest X-ray). 

Individuals aged ≥65 years were considered in the study as the focus population. We considered as a reference population, for comparison purposes, the included younger adults (<65 years old). 

### 2.1. Clinical Variables, Data Source, and Study Outcomes

Demographic and clinical data, as well as the information from the episode (laboratory workup, electrocardiogram, vital signs, treatment), were extracted from the electronic medical record using standardized data collection. Myocardial injury was defined considering sex-specific cutoff for hs-TnT value greater than the institutional upper limit of normal, i.e., 9 ng/L for female and 16 ng/L for male, without electrocardiographic changes that suggest acute ischemia, nor any other acute heart condition [5]. Clinical severity was assessed at admission with MEWS score [10,11]. Laboratory workups were systematized with an at-admission protocol that included a blood draw with full blood count, electrolytes, renal and liver function, cardiac biomarkers (high-sensitivity troponin T (hs-TnT), N-terminal-proB-type natriuretic peptide (NT-proBNP) and lactate dehydrogenase (LDH)), inflammatory markers (C-reactive protein (C-RP), interleukin-6 (IL-6), serum ferritin, and coagulation testing (D-dimer). Comorbidity was assessed using Charlson Comorbidity Index [12], a widely used index that is comprised of 19 comorbid conditions: myocardial infarct, congestive heart failure, peripheral vascular disease, cerebrovascular disease, dementia, chronic pulmonary disease, connective tissue disease, ulcer disease, mild liver disease, diabetes, hemiplegia, moderate or several renal disease, diabetes with end organ damage, any tumor, leukemia, lymphoma, moderate or severe liver disease, metastatic solid tumor, and AIDS. Each disease is given a different weight based on the strength of its association with 1-year mortality. For classification purposes, we categorized in the following: no comorbidities, mild (1–2 comorbidities), and severe (≥3 comorbidities). 

Key outcomes included time to clinical stability (defined as the time the time elapsed since the patient’s admission to all of the following: oxygen saturation > 94% (FiO_2_ 21%), normalized level of consciousness (baseline), HR < 100 rpm, systolic BP > 90 mm Hg, Temperature < 37.2 °C), admission to a critical care unit, or in-hospital mortality.

### 2.2. Ethics Considerations

The Institutional Ethics Committee of Hospital del Mar of Barcelona approved the study and, due to the nature of the retrospective data review, waived the need for informed consent from individual patients (CEIm 2020/9352).

### 2.3. Statistical Analysis

Continuous variables are expressed as medians and interquartile range (IQRs). Categorical variables are expressed as frequencies (percentages). Continuous variables were compared using the Student t-test or the Mann–Whitney U test, as appropriate, and categorical variables using χ^2^ test or the Fisher exact test, as appropriate. 

Spearman correlation was used to test the association between inflammatory markers, and pro-NT-BNP and hs-TnT. 

A logistic regression model was fitted to determine odd ratios (ORs) and 95% confidence intervals (CI) for covariates with in-hospital mortality as outcome. We considered age and gender, comorbidities (pre-existing coronary artery disease, hypertension, diabetes mellitus or cerebrovascular disease), severity of the episode (MEWS score) and days with symptoms before admission as predictive variables in the model. All statistical analyses were performed using STATA/MP V.14.0, and a two-sided *p* value of <0.05 was considered statistically significant.

## 3. Results

**Figure 1:**
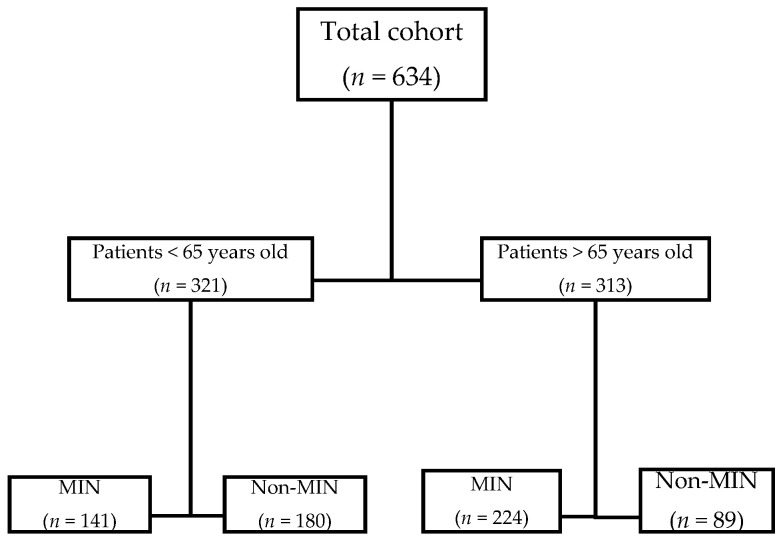
Flowchart of the patients included in the study.

Among 634 patients admitted during the period of study, 365 had MIN (58%). When divided by age, 313 (49%) were older adults, and, of them, 224 (61%) presented with MIN. The population and subgroups study are shown in Figure 1. Baseline characteristics of older adults are shown in Table 1 and in Table S1 (Supplementary Materials).

Differences among older adults and younger adults with MIN were found (Table S2). In brief, individuals in the older group with MIN were more likely to have cardiovascular risk factors (hypertension 78% versus 21%; *p* < 0.001), diabetes mellitus (33% versus 8%; *p* < 0.001), or chronic heart disease (30% versus 4.5%; *p* < 0.001).

Interestingly, there were also differences in the clinical presentation, since dyspnea was more frequently the main symptom among the older adults (53% versus 34%; *p* < 0.001), and they also presented higher inflammatory markers, such as C-RP (8.68 mg/dL versus 6.2 mg/dL; *p* = 0.02) and significantly higher NT-ProBNP (861UI/l versus 162UI/l; *p* = 0.007). Along with these differences, we found that older adults with MIN were more prone to die during hospitalization (31% versus 3%; *p* < 0.001) (Table S2).

When focusing only in the older adults group with MIN, they were more likely to be older (mean age of 83 years (75–88) versus 74 (68–78); *p* < 0.001) and male (48% versus 33%; *p* = 0.02), with higher prevalence of cardiovascular disease (diabetes mellitus, hypertension, heart disease) and with no differences in duration of symptoms at admission (Table 1). Remarkbly, MIN was associated with higher mortality rates (71 (31%) versus 11 (12%); *p* < 0.001). 

There was a significant correlation between hs-TnT at admission and IL-6 levels (Spearman’s Rho 0.201; *p* = 0.028) or C-RP (Spearman’s Rho: 0.251; *p* = 0.001). In addition, a positive significant correlation between hs-TnT and NT-ProBNP was found (Spearman’s Rho = 0.593; *p* < 0.001), showing a potential association between myocardial injury and myocardial dysfunction. 

When comparing both groups, those with MIN had significantly higher inflammatory markers at admission compared to those without MIN, such as C-RP (median 8.68 mg/dL (IQR 3.8–18.6) versus 5.3 mg/dL (IQR 2.3–11.2); *p* = 0.02), lower lymphocyte count (median count 0.88/mL (0.62–1.29) versus 1.07/mL (0.82–1.63); *p* = 0.001), and higher levels of D-dimer (median 1215UI/l (680–2540) versus 780 UI/l (450–1330); *p* < 0.001) (Table 1). 

MIN showed longer time to clinical recovery compared to those without it (median 13 days (IQR 6–21) versus 9 days (IQR 5–17); *p* < 0.001). In-hospital mortality was also significantly higher in the MIN group (71 (31%]) versus 11 (12%); *p* < 0.001).

In a logistic regression model adjusting by age, sex, severity, and Charlson Comorbidity Index, the OR for in-hospital mortality was 2.1 (95% CI 1.02–4.42; *p* = 0.043) among those older adults with myocardial injury at admission (Table 2).

## 4. Discussion

We report a significant association between MIN in older adults and worse prognosis consisting in prolonged time to recovery and higher mortality rates, as reported in similar smaller series [6,7,13,14]. 

This study adds above and beyond recent similar studies, underscoring the importance of myocardial injury as an independent prognostic factor. Individuals with MIN were more frequently male, have more diabetes, and have more chronic heart disease, but, after controlling for these possible confounders, MIN was still associated with worse clinical prognosis. These are well-established risk factors for adverse events in outbreaks of respiratory virus infections [15,16]. However, similarly, Case et al. reported that MIN was associated with worse prognosis and higher requirement of mechanical ventilation [7]. In another similar series, Nuzzi et al. also reported the relevance of a systematic evaluation of myocardial injury, not only at admission but also within the first 48 h of in-hospital stay, due to its implication on prognosis [13]. 

The prevalence of MIN varies, depending on the type of patients studied. We found that MIN was present in 50% of our overall cohort, and up to 70% when looking into older adults. However, in other studies, MIN has been reported as a less frequent condition, presented in wide range from 10% to more than 50% of patients with COVID-19 [7,16,17,18,19,20]. The higher rates observed in this study might be due to only consider individuals requiring hospitalization that, during the first wave, were older and more comorbid [12]. Other prior studies included mixed series with inpatients and outpatients with different SARS-CoV-2 severity [18]. When considering series with hospitalized individuals, only the MIN prevalence was more similar to ours, ranging from 60 to 75% [6,14,21]. 

This study is focused on older adults, as a population that globally suffered more casualties during the first wave of the pandemic, and adds specific evidence to prior reports [6,7,13]. Older adults showed significantly higher mortality rates than those younger counterparts, showing that the differences are, probably, not only because of MIN but, rather, due to other comorbidities besides age. 

SARS-CoV-2 shows high tropism by myocardial cells due to the ability of the virus to infect the myocardium through its binding to angiotensin II converting enzyme II (ACE2) highly expressed by myocardial cells [22]. During acute infection, it is likely that an impairment on heart function will take place. In fact, myocardial injury has been associated in other series with myocardial dysfunction [3]. In our series, we found that individuals presenting with myocardial injury at admission more frequently had heart failure, both by clinical and biochemical signs measured by NT-proBNP. Unfortunately, we were not able to measure ejection fraction during the episode, due to unbelievable pressure on healthcare in the acute moment of the pandemic. Likewise, with our data, we are inclined to assess that there is an impairment of function, as well. 

There is a wide range of hypotheses trying to discern the causes of troponin rise during SARS-CoV-2 infection, as well as the role it plays in the evolution of the disease [8]. An interesting hypothesis is that cardiac injury may reflect an ongoing pathological insult due to inflammation or secondary to hypoxemia [23]. But, in our series, we found no differences between older adults with or without MIN in terms of the ratio of arterial oxygen partial pressure to fractional inspired oxygen (PaFi) at admission, moving us away from the theory that MIN is directly related to hypoxemia. 

Conversely, according to our results, we entertain the hypothesis that inflammatory pathways put in place by the immune response against the virus collaterally affect the myocardium. We found a positive correlation between inflammation measured by inflammatory markers and troponin levels. In addition, we consistently found that individuals with MIN showed significantly higher inflammatory markers than those individuals without it. Older adults might be more susceptible to increased inflammation in the context of a viral infection as a result of a process called immunosenescence. This consists of the aging of the immune system [24] that leads to abnormal immune responses [3], due to a specific shaping of the immune system inducing more inflammation [19], driving an age-related increased response against pathogens [19]. Therefore, the convergence of a tissue highly infected by the virus [8] and abnormal inflammatory response [19] against it might, at least, partially, explain the higher incidence and worse outcomes of MIN among older adults. 

Our study has some limitations since it was conducted in a single center during the first wave of the COVID-19 outbreak. It has a mid-size sample, and we were not able to conduct cardiac ultrasound or MRI; as a consequence, we were only able to define myocardial injury by troponin elevation, without detailing myocardial tissue characteristics and hemodynamic function. Moreover, during this first wave, many older adults with SARS-CoV-2 infection remained in long term care facilities. In that case, we can say that our hospital was the reference center for many long-term care facilities of the area and received frequent referrals from them, even in the moments with the highest incidence. 

This study was conducted during the first wave of the pandemic, where individuals had no prior contact with the virus, and the immune response was naïve. The impact of vaccination in this condition, especially its incidence or its prognosis value, remains to be elucidated in the new scenario and might constitute future research. 

However, we find that a key strength of our study is that it is a representative sample of an older adult population, belonging to a large city with high incidence of SARS-CoV-2 infection, which makes the results directly relevant to the clinical practice. 

## 5. Conclusions

We can conclude that MIN was frequent in older adults with SARS-CoV-2 infection, especially in patients with pre-existing comorbidities and with higher inflammatory levels. We can also conclude that MIN impacted the clinical outcomes of individuals that experienced it, being associated with greater time to clinical recovery, more severe presentation of the disease, and higher odds of in-hospital mortality. The consistent association that we found between inflammation and MIN makes the hypothesis of an inflammatory insult as responsible for heart damage plausible. This is more relevant in a group with higher inflammatory levels due to immune dysregulation linked to aging and may deserve further attention. Ultimately, due to its widespread presence, and its likely role in prognosis, it is advisable that we direct attention to this matter.

## Figures and Tables

**Table 1:** Baseline characteristics and comparison between individuals with and without myocardial injury.

	Overall		Myocardial Injury		Without Myocardial Injury		*p*-Value
Cohort Characteristics	*n* = 313			*n* = 224	*n* = 89		
Median age, years (IQR)	79	(73–87) *	83	(75–88)	74	(68–78)	<0.001
Male sex (%)	134	(44%)	105	(48%)	29	(33%)	0.022
Long Term Care Facility (%)	59	(19%)	47	(21%)	12	(13%)	0.231
Dependent for life activities (%)	42	(14%)	39	(18%)	3	(3%)	0.002
Comorbidities							
Current smoker (%)	11	(3.5%)	9	(4%)	2	(2.2%)	0.442
Hypertension (%)	230	(74%)	175	(78%)	55	(62%)	0.033
Diabetes Mellitus (%)	87	(28%)	75	(33%)	12	(13%)	<0.001
Chronic lung disease (%)	31	(10%)	25	(11%)	6	(7%)	0.238
Chronic heart disease (%)	78	(25%)	71	(32%)	7	(8%)	<0.001
Chronic renal disease (%)	88	(28%)	66	(29%)	22	(24%)	0.400
Chronic liver disease (%)	17	(%)	13	(6%)	4	(4.5%)	0.645
Dementia (%)	54	(17%)	45	(20%)	9	(3%)	0.035
ARB-2 (%)	57	(18%)	47	(21%)	10	(11%)	0.050
ACE inhibitors (%)	98	(31%)	70	(31%)	28	(31%)	0.971
Estatins (%)	116	(37%)	83	(37%)	33	(37%)	0.997
Charlson Comorbidy Index, median (IQR)							
No comorbidity, *n* (%)	81	(25%)	41	(21%)	40	(45%)	<0.001
Medium-low (1–2), *n* (%)	104	(33%)	76	(34%)	28	(31%)	0.416
High (≥3), *n* (%)	128	(42%)	107	(45%)	21	(24%)	<0.001
Onset symptoms							
Dyspnoea (%)	158	(50%)	119	(53%)	39	(44%)	0.137
Fever (%)	222	(71%)	146	(65%)	76	(85%)	<0.001
Cough (%)	203	(65%)	131	(58%)	72	(81%)	<0.001
Consciousness impairment (%)	58	(18%)	52	(23%)	6	(7%)	0.001
Confirmed Pulmonary Emboslism (%)	15	(5%)	9	(4%)	6	(7%)	0.309
Acute Abnormalities in the EKG (%)	13	(4%)	12	(5%)	1	(1%)	0.090
Clinical markers at onset							
Median C-Reactive Protein mg/dL (IQR)	7.3	(3.3–15.4)	8.68	(3.8–18.6)	5.3	(2.3–11.2)	0.02
Procalcitonin mg/dL (IQR)	0.152	(0.09–0.36)	0.21	(0.10–0.54)	0.10	(0.07–0.17)	0.02
Median lymphocyte count /mL (IQR)	0.955	(0.65–1.4)	0.88	(0.62–1.29)	1.07	(0.82–1.63)	0.001
Median IL-6 pg/mL (IQR)	49	(19–103)	45	(12–131)	57	(25–85)	0.112
Median Lactate Dehydrogenase UI/l (IQR)	285	(232–386)	288	(232–411)	271	(236–344)	0.038
Median D-Dimer UI/l (IQR)	1000	(620–2200)	1215	(680–2540)	780	(450–1330)	<0.001
Median Pro-BNP UI/l (IQRS)	487	(222–1391)	861	(344–3316)	235	(101–349)	0.001
Median Creatinin mg/dl(IQR)	0.99	(0.77–1.26)	1.08	(0.85–1.47)	0.79	(0.67–0.95)	<0.001
Median PaFi (IQR)	180	(95–289)	166	(91–281)	219	(101–310)	0.213
Median MEWS (IQR)	2	(1–3)	2	(2–3)	2	(1–2)	0.004
Median Cholesterol mg/mL (IQR)	134	(118–161)	142	(119–162)	131	(113–143)	0.072
Clinical Outcomes							
Median Time to clinical recovery days (IQR)	12	(6–20)	13	(6–21)	9	(5–17)	0.036
ICU admission (%)	46	(15%)	29	(14%)	17	(19%)	0.230
Death (%)	82	(26%)	71	(31%)	11	(12%)	<0.001

* IQR: interquartile Range; ARB-2: Angiotensin II receptor blockers; ACE: Angiotensin-converting enzyme; EKG: Electrocardiogram.

**Table 2:** Logistic multivariable regression. Predictors of in-hospital mortality.

	Odds-Ratio	95% CI	*p*-Value
Myocardial Injury	2.1	1.02-4.42	0.043
Age	1.08	1.05-1.11	<0.001
Sex	1.11	0.98-1.32	0.093
Charlson Index	1.11	1.03-1.19	0.004
MEWS	1.125	1.01-1.31	0.019

MEWS = severity score at admission (Modified Early Warning Score).

## Data Availability

We have not planned to upload our data for sharing. This data come from a general database that is being collected in real time information about all the admissions with SARS-CoV-2 infection in the hospital. However, datasets are available from the corresponding author on reasonable request.

## Supplementary Materials

The following are available online at https://www.mdpi.com/article/10.3390/jcm10163738/s1, Table S1: Main clinical characteristics of the cohort. Differences between older adults with and without Myocardial Injury. Table S2: Comparison between younger and older adults with Myocardial Injury.
