# Supplementary material for: Prevalence and Prognostic Value of Myocardial Injury in the Initial Presentation of SARS-CoV-2 Infection among Older Adults
Source: J Clin Med. 2021 Aug 23;10(16):3738. doi: 10.3390/jcm10163738 (PMC8397085; doi:10.3390/jcm10163738)
Supplement: Supplementary file 1 [file jcm-10-03738-s001.zip › jcm-1293137-supplementary.pdf]

Table S1. Baseline characteristics. And comparison between individuals with and without myocardial injury

	Overall		*Myocardial Injury*		*Without Myocardial Injury*		*p-value*
**Cohort Characteristics**	n=313		n=224		n=89		
Median age, years (IQR)	79	(73-87)*	83	(75-88)	74	(68-78)	**<0.001**
Male sex (%)	134	(44%)	105	(48%)	29	(33%)	**0.022**
Long Term Care Facility (%)	59	(19%)	47	(21%)	12	(13%)	0.231
Partially Dependent for life activities (%)	62	(21%)	52	(24%)	10	(11%)	**0.024**
Totally Dependent for life activities (%)	42	(14%)	39	(18%)	3	(3%)	**0.002**
**Comorbidities**							
Current smoker (%)	11	(3.5%)	9	(4%)	2	(2.2%)	0.442
Hypertension (%)	230	(74%)	175	(78%)	55	(62%)	0.033
Diabetes Mellitus (%)	87	(28%)	75	(33%)	12	(13%)	**<0.001**
Chronic lung disease (%)	31	(10%)	25	(11%)	6	(7%)	0.238
Chronic heart disease (%)	78	(25%)	71	(32%)	7	(8%)	**<0.001**
Chronic renal disease (%)	88	(28%)	66	(29%)	22	(24%)	0.400
Chronic liver disease (%)	17	(%)	13	(6%)	4	(4.5%)	0.645
Dementia (%)	54	(17%)	45	(20%)	9	(3%)	**0.035**
Autoimmunitary Disease (%)	14	(4%)	9	(4%)	5	(5.5%)	0.537
HIV-infection (%)	1	(0.3%)	1	(0.5%)	0	(0%)	0.669
Obesity (%)	57	(18%)	40	(18%)	17	(19%)	0.797
Active cancer (%)	13	(4%)	12	(5%)	1	(1%)	0.090
ARB-2 (%)	57	(18%)	47	(21%)	10	(11%)	**0.050**
ACE inhibitors (%)	98	(31%)	70	(31%)	28	(31%)	0.971
Estatins (%)	116	(37%)	83	(37%)	33	(37%)	0.997

Charlson Comorbidy Index, median (IQR)							
No comorbitiy, n (%)	81	(25%)	41	(21%)	40	(45%)	**<0.001**
Medium-low (1-2), n (%)	104	(33%)	76	(34%)	28	(31%)	0.416
High (≥3), n (%)	128	(42%)	107	(45%)	21	(24%)	**<0.001**
**Onset symptoms**							
Dyspnoea (%)	158	(50%)	119	(53%)	39	(44%)	0.137
Fever (%)	222	(71%)	146	(65%)	76	(85%)	**<0.001**
Cough (%)	203	(65%)	131	(58%)	72	(81%)	**<0.001**
Diarrhoea (%)	74	(24%)	48	(21%)	26	(29%)	0.144
Headache (%)	22	(7%)	13	(6%)	9	(10%)	0.179
Anosmia (%)	16	(5%)	7	(3%)	9	(10%)	0.011
Consciousness impairment (%)	58	(18%)	52	(23%)	6	(7%)	**0.001**
Confirmed Pulmonary Emboslism (%)	15	(5%)	9	(4%)	6	(7%)	0.309
Acute Abnormalities in the EKG (%)	13	(4%)	12	(5%)	1	(1%)	0.090
**Clinical markers at onset**							
Median C-Reactive Protein mg/dl (IQR)	7.3	(3.3-15.4)	8.68	(3.8-18.6)	5.3	(2.3-11.2)	**0.02**
Procalcitonin mg/dl (IQR)	0.152	(0.09-0.36)	0.21	(0.10-0.54)	0.10	(0.07-0.17)	**0.02**
Median lymphocyte count /ml (IQR)	0.955	(0.65-1.4)	0.88	(0.62-1.29)	1.07	(0.82-1.63)	**0.001**
Median IL-6 pg/ml (IQR)	49	(19-103)	45	(12-131)	57	(25-85)	0.112
Median Lactate Dehydrogenase UI/l (IQR)	285	(232-386)	288	(232-411)	271	(236-344)	**0.038**
Median D-Dimer UI/l (IQR)	1000	(620-2200)	1215	(680-2540)	780	(450-1330)	**<0.001**
Median Pro-BNP UI/l (IQRS)	487	(222-1391)	861	(344-3316)	235	(101-349)	**0.001**
Median Troponin UI/l (IQR)							
Median Creatinin mg/dl(IQR)	0.99	(0.77-1.26)	1.08	(0.85-1.47)	0.79	(0.67-0.95)	**<0.001**
Median PaFi (IQR)	180	(95-289)	166	(91-281)	219	(101-310)	0.213

Median MEWS (IQR)	2	(1-3)	2	(2-3)	2	(1-2)	**0.004**
Median Cholesterol mg/ml (IQR)	134	(118-161)	142	(119-162)	131	(113-143)	0.072
**Treatment**							
Hydroxycholoroquine (%)	266	(89.5%)	183	(86%)	83	(95%)	**0.028**
Azythromicin (%)	269	(90%)	185	(87%)	84	(96%)	**0.019**
Tocilizumab (%)	35	(12%)	18	(8.5%)	17	(19%)	**0.008**
Dexamethasone (%)	61	(19%)	47	(21%)	14	(15%)	0.432
Methylprednisolone (%)	69	(22%)	52	(23%)	17	(19%)	0.428
**Clinical Outcomes**							
Median Time to clinical recovery days (IQR)	12	(6-20)	13	(6-21)	9	(5-17)	**0.036**
ICU admission (%)	46	(15%)	29	(14%)	17	(19%)	0.230
Death (%)	82	(26%)	71	(31%)	11	(12%)	**<0.001**

Table S2. Comparison between younger adults ant older adults with Myocardial Injury

	Younger adults (n=321)		*Older Adults (n=313)*		*p-value*
Myocardial Injury	N=141 (43%)		N=224 (71%)		<0.001
Median age, years (IQR)	51	(42-58)	83	(75-88)	**<0.001**
Male sex (%)	60	(43%)	105	(47%)	0.432
Barthel index at admission, median (IQR)	100	(100-100)	95	(51-100)	**<0.001**
Long Term Care Facility (%)	2	(1%)	47	(20%)	**<0.001**
Partially Dependent for life activities (%)	6	(4%)	10	(23%)	**<0.001**
Totally Dependent for life activities (%)	3	(2%)	39	(17%)	**<0.001**

**Comorbidities**					
Current smoker (%)	19	(14%)	9	(4%)	**0.001**
Hypertension (%)	30	(21%)	175	(78%)	**<0.001**
Diabetes Mellitus (%)	12	(8%)	75	(33%)	**<0.001**
Chronic lung disease (%)	6	(4%)	25	(11%)	**0.021**
Chronic heart disease (%)	7	(4.5%)	71	(31%)	**<0.001**
Chronic renal disease (%)	99	(70%)	66	(29%)	**<0.001**
Chronic liver disease (%)	5	(3%)	13	(6%)	0.335
Dementia (%)	2	(1.5%)	45	(20%)	**<0.001**
Autoimmunitary Disease (%)	4	(3%)	9	(4%)	0.553
HIV-infection (%)	4	(3%)	1	(0.5%)	**0.002**
Obesity (%)	19	(13%)	40	(18%)	0.268
Active cancer (%)	1	(1%)	12	(8%)	0.090
ARB-2 (%)	7	(5%)	47	(21%)	**<0.001**
ACE inhibitors (%)	13	(9%)	70	(31%)	**<0.001**
Estatins (%)	13	(9%)	83	(37%)	**<0.001**
Charlson Comorbidy Index, median (IQR)	0	(0-1)	2	(1-4)	**<0.001**
No comorbitiy, n (%)	108	(74%)	41	(18%)	**<0.001**
Medium-low (1-2), n (%)	18	(11%)	79	(35%)	**<0.001**
High (≥3), n (%)	21	(15%)	104	(47%)	**<0.001**
**Onset symptoms**					
Dyspnoea (%)	49	(34%)	119	(53%)	**<0.001**
Fever (%)	87	(61%)	146	(65%)	0.532
Cough (%)	89	(63%)	131	(58%)	0.378
Diarrhoea (%)	29	(20%)	48	(21%)	0.844
Headache (%)	28	(20%)	13	(6%)	**<0.001**
Anosmia (%)	43	(30%)	7	(3%)	**0.011**
Consciousness impairment (%)	5	(3%)	52	(23%)	**<0.001**
Confirmed Pulmonary Emboslism (%)	4	(3%)	9	(4%)	0.553
Acute Abnormalities in the EKG (%)	2	(1.5%)	12	(5%)	0.090

**Clinical markers at onset**					
Median C-Reactive Protein mg/dl (IQR)	6.2	(3.8-18.6)	8.68	(3.8-18.6)	**0.02**
Procalcitonin mg/dl (IQR)	0.22	(0.12-0.56)	0.21	(0.10-0.54)	0.274
Median lymphocyte count /ml (IQRS)	1.21	(0.82-1.53)	0.88	(0.62-1.29)	0.434
Median IL-6 pg/ml (IQR)	58	(20-115)	45	(12-131)	0.502
Median Lactate Dehydrogenase UI/l (IQR)	274	(212-430)	288	(232-411)	0.721
Median D-Dimer UI/l (IQR)	800	(430-1470)	1215	(680-2540)	0.276
Median Pro-BNP UI/l (IQRS)	162	(134-528)	861	(344-3316)	**0.007**
Median Troponin UI/l (IQR)	24	(18-41)	30	(20-54)	0.122
Median Creatinin mg/dl(IQR)	0.91	(0.75-1.27)	1.08	(0.85-1.47)	0.415
Median PaFi (IQR)	188	(106-310)	166	(91-281)	0.299
Median MEWS (IQR)	2	(1-3)	2	(2-3)	0.316
Median Cholesterol mg/ml (IQR)	145	(108-168)	142	(119-162)	0.810
**Treatment**					
Hydroxycholoroquine (%)	72	(53%)	183	(86%)	**<0.001**
Azythromicin (%)	75	(55%)	185	(87%)	**0.019**
Tocilizumab (%)	16	(12%)	18	(8.5%)	0.318
Dexamethasone (%)	13	(9%)	47	(21%)	**0.003**
Methylprednisolone (%)	15	(12%)	52	(23%)	**0.002**
**Clinical Outcomes**					
Median Time to clinical stability days (IQR)	11	(6-26)	13	(7-21)	0.936
ICU admission (%)	23	(17%)	29	(14%)	0.397
Mechanical Ventilation	21	(15%)	45	(20%)	0.223
Death (%)	4	(3%)	71	(31%)	**<0.001**
